# A global perspective on decadal challenges and priorities in biodiversity informatics

**DOI:** 10.1186/s12898-015-0046-8

**Published:** 2015-05-29

**Authors:** A Townsend Peterson, Jorge Soberón, Leonard Krishtalka

**Affiliations:** Biodiversity Institute, University of Kansas, 1345 Jayhawk Blvd., Lawrence, KS 66045 USA

**Keywords:** Biodiversity informatics, Data, Infrastructure, Training, Capacity-building

## Abstract

Biodiversity informatics is a field that is growing rapidly in data infrastructure, tools, and participation by researchers worldwide from diverse disciplines and with diverse, innovative approaches. A recent ‘decadal view’ of the field laid out a vision that was nonetheless restricted and constrained by its European focus. Our alternative decadal view is global, i.e., it sees the worldwide scope and importance of biodiversity informatics as addressing five major, global goals: (1) mobilize existing knowledge; (2) share this knowledge and the experience of its myriad deployments globally; (3) avoid ‘siloing’ and reinventing the tools of knowledge deployment; (4) tackle biodiversity informatics challenges at appropriate scales; and (5) seek solutions to difficult challenges that are strategic.

## Background

Biodiversity informatics (BI) is simultaneously an old field and a very young one. Its major sources of data are old: records associated with physical voucher specimens housed in museums and herbaria that, in many cases, are still in the form of cross-referenced card files, paper catalogs, and other pre-digital ledgers. As a new discipline, however, BI has a computer-aided history of only a few decades, evolving from simple databases of collections and observations to detailed, interactive, and flexible systems of information management, modeling, analysis, and interpretation. Indeed, BI as a research enterprise in terms of analytical and theoretical power, sophistication, and research output, has expanded enormously during the last two decades.

Several workers in the field, however, have expressed concern that this arena of research is not driven by conceptual inquiry and fundamental questions. For example, a recent analysis [1] concluded that developments in BI have been driven largely by availability of technologies and data, and rarely by important and exciting conceptual challenges and theoretical predictions. That is, BI’s evolution to date has been driven by the kinds of inquiry that become tractable or feasible, rather than by grand challenge questions that seek to discover deep, underlying patterns and processes: e.g., how many species inhabit Earth and what processes govern their distributions? Such key questions have largely lain fallow.

Hardisty and Roberts [2] laid out a ‘decadal view’ of challenges and priorities in BI, with several goals that are sound and that we applaud. However, their viewpoint looks solidly northward, i.e., their BI world is explicitly and almost exclusively European. It is highly commendable that the European community advances its BI resources and capabilities. However, biodiversity, which is richest in the Tropics, is a global phenomenon: the majority of species are on other continents, as are the bulk of biodiversity scientists and users of the science. Finally, as many others have noted, northern institutions, including European museums and herbaria, hold much of the historical, legacy biodiversity information—voucher specimens and associated data—for many of the Tropical countries, owing to colonial-era explorations. Indeed, in this sense, the rest of the world requires and depends on advances in European BI, but ideally these efforts should be informed, designed, mediated, and implemented by a global view, one framed in international and intercontinental contexts.

This communication offers an alternative decadal view for biodiversity informatics. Hardisty and Roberts [2] listed tasks that have largely already been initiated or, in some cases, resolved. A more profound and challenging set of tasks lies ahead: (a) capture data associated with the billions of biodiversity information records (i.e., scientific specimens) held in ‘northern’ museums and herbaria; (b) share those data efficiently and collaboratively, effectively repatriating the data to countries of origin; and (c) share investment in training new generations of scientists in the concepts, tools, and theory to model, analyze, and apply these vast new data resources. Accomplishing these three tasks will propel BI worldwide, and will create a potent force in the overriding goal of informing and advancing smart global environmental stewardship.

## Biodiversity-rich and (frequently) information-poor regions

The countries and regions of the Earth are characterized by marked differences in richness of biodiversity. Specifically, among well-known biodiversity gradients, the temperate-to-tropical one is dominant, with tropical regions holding biotas that are considerably more diverse. This imbalance links to the Linnaean and Wallacean shortfalls [3, 4], which, respectively, are the massive gaps in knowledge about the details of the diversity and distribution of units of biodiversity. These gaps are particularly acute in the developing world, where biodiversity tends to be understudied in spite of its richness, and for which the huge volume of existing biodiversity data is still not available.

**Figure 1:**
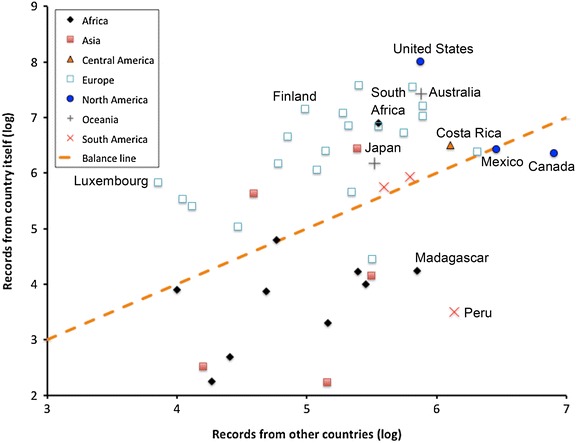
Summary of Digital Accessible Knowledge for countries worldwide, drawn from the Global Biodiversity Information Facility in January 2014, showing log_10_ of numbers of records coming from the within the country versus those being provided by institutions in other countries. Countries (many, from all continents) that serve no data are omitted from the graphic. The *dashed line* indicates even balance between records from inside and outside of the country.

In sharp contrast to this biodiversity gradient is the reverse pattern of the history and current status of the world’s wealth, power, and education, and its collateral effect of much less access to information and education. Colonial history, among other factors, particularly in Tropical regions during the period of most intense biodiversity exploration (approximately 1850–1950), resulted in massive collections of animals and plants and associated data being extracted from these countries and deposited in institutions across Europe and North America (Figure 1). This bias is mirrored by the demographics of biodiversity specialists, who are similarly concentrated in North American and European institutions [5].

Now, however, the geography of the biodiversity science enterprise is in rapid flux, with strong growth in research, education, and infrastructure since the end of the twentieth century in many developing countries [6]. Indeed, many sectors of the developing world—most notably Mexico, Colombia, Brazil, and South Africa—have achieved such growth that they have ‘flattened’ the world of global biodiversity science; several other countries are not far behind. As such, this globalization of BI resources, expertise, and research is redefining and broadening the ‘centers’ of the biodiversity science universe to domains beyond North America and Europe; the process is far from complete, but the tendency is clear.

## The uneven state of biodiversity science in Europe

Biodiversity science in Europe is thriving. Numerous research groups are generating systematic revisions (e.g., [7]), molecular phylogenetic and phylogeographic studies (e.g., [8]), biogeographic and ecological models (e.g., [9]), and environmental syntheses [10]. Other initiatives are extending biodiversity science in Europe to related fields (e.g., B4Life, BEST, EBRI).

Simultaneously, however, the underlying promise of future European biodiversity science might be seriously constrained by institutional history and culture. Breakthrough advances in biodiversity science depend on harnessing and integrating two primary realms of evidence: one comprises legacy biodiversity data, such as those documented by existing biocollections in museums and herbaria; the second realm comprises data from new, rich, and geographically widespread biocollections that are focused by modern research questions. With some taxonomic and institutional exceptions [11], European biocollections appear to be failing both sides of this critical equation: the legacy collections, despite their overwhelming importance in documenting past global biodiversity [12], are not being digitized or shared at a rate that will bring them into currency for science and society in time to inform solutions to the planet’s biodiversity crisis (note, e.g., that the Natural History Museum of the UK appears to serve no records via GBIF; the Royal Botanic Gardens of the UK serves only 728,527 records out of a total of 7 million specimens, or about 10%) [13]. At the same time, the impetus is modest, if not absent, for conducting new, collections-based surveys and inventories that document current global biodiversity with new methodologies and tools, even within Europe [14]. As such, and again with exceptions, European biocollections institutions are neither investing in the future of BI, nor evolving the BI potency of the enormous volume of data already resident in their museum cases and ledgers.

Instead, Europe appears to be a champion of biodiversity meetings, workshops, and conferences (e.g., the recent e-Biosphere and GBIC [15] congresses), the vast majority of which merely repeat the points and priorities from decades of previous meetings, and conclude, as action items, the need for more meetings. Of course, European institutions are not alone in this malaise, but the situation there appears to be more acute than in the Americas, Asia, Africa and Australia, where institutions are more actively grabbing the BI future.

## Challenges and priorities

Of the detailed list of recommendations by Hardisty and Roberts [2], many appear to be post hoc and anticipatory of activities already begun. Essentially, their decadal view is of the past decade, not the next one, thus promising little new in the way of progress. For example:

1. Their challenge to assemble a comprehensive taxonomic summary of biodiversity does not seek a compendium of valid taxonomic names, but merely a list of names in use, a much more modest goal.
2. Their recommendation to develop persistent identifiers for biodiversity records has been a consistent topic of intense discussion and development [16–19] during the past decade; moreover, persistent identifiers are already in use in many institutions.
3. Their recommendation for mechanisms to evaluate data fitness for use in biodiversity studies misses major advances and effective solutions already in place [20, 21].
4. Their call to address the management and integration of observational data is apparently unaware of substantial accomplishments in this arena by the AudubonCore group [22].

These and other examples from their paper [2] illustrate a vision of slow, gradual, incremental change, often in areas in which significant change has already begun or occurred. Perhaps the most serious casualty of this incremental view are Europe’s vast legacy collections and associated data records of past global biodiversity. With exceptions, digitization of this huge library of biodiversity information is either not occurring, or is occurring too slowly and haphazardly for global biodiversity science to progress. Indeed, digital mobilization of existing biodiversity knowledge is the first of five challenges we offer for the next decade. These challenges are designed to be fully global, applying equally across the international community of biodiversity institutions and infrastructures.

### Challenge #1: Mobilize existing knowledge

Biocollections of scientific specimens are, in effect, massive storehouses of irreplaceable biodiversity data. Although data aggregated from heterogeneous sources often have problems [23–28], such data are used extensively and increasingly by scientists in both developed and developing countries. For example, in 2013, the Global Biodiversity Information Facility (GBIF) data portal saw >130,000 visits from locations in the United States, but with many thousands of visits from Mexico, Colombia, Argentina, Brazil, and India, among many others; indeed, GBIF’s new data portal (2013) served 3.67 billion records in its first 40 h [29]. As of its last report (August 2014), GBIF has compiled 886 scientific papers that used GBIF-mediated data in analyses ranging from basic research to applications of biodiversity policy (http://www.gbif.org/mendeley/usecases); although surely some of those papers do not actually use GBIF-mediated data in analyses, the utility of the resource is clear.

However, the enormous volume of biodiversity data that remains in analog format is nowhere near as easily accessed, shared, analyzed, or interpreted. Progress in accelerating and optimizing workflows and protocols for digitizing such data has demonstrated that meeting this challenge is increasingly feasible [30, 31]. Recent estimates are that museums and herbaria worldwide hold 1.5–2.0 billion specimens [32, 33], yet only about 10% of that total is currently accessible via GBIF, the largest aggregator of specimen records. Although smaller-scale initiatives provide access to additional specimen records (an excellent example is speciesLink, http://www.splink.org.br/), the bulk of the data associated with the world’s biocollections remain inaccessible to biodiversity science.

The causes for this lack of progress in digitization and sharing of biocollections data are manifold, but common themes are budgetary and sociological [30]. Data ‘owners’ cite a spectrum of reasons: fear of activists or biopiracy; concern about insufficient data quality; a desire for economic return, or to control access and use of the scientific data; and the cost of digitizing collection data, on the order of US$1–10 per specimen [34], although initiatives have begun to reduce these costs significantly (http://beyondthebox.aibs.org/). Another possibility is that institutions may have assessed costs and benefits of such efforts, and decided that digitization is not worth the effort and investment. In many cases, however, the most serious hurdle is simply institutional inertia or strategic apathy—digital mobilization of their collections data is not a priority.

Even when collections data are in digital format, they often are not made available broadly and openly, despite major community technological initiatives to foster data access and sharing including development of the DarwinCore standard, information transfer protocols such as DiGIR and IPT, and implementation of large-scale biodiversity information portals (e.g., VertNet, GBIF, speciesLink, REMIB, UNIBIO, SEINet, iDigBio).

Whatever the reasons, in effect, by not moving ahead in digitizing data, institutions effectively quarantine and sequester biodiversity knowledge held in non-digital formats from modern research on biodiversity phenomena of considerable interest and currency. Rescuing these data digitally from stealth mode enables biodiversity informatics to transform a descriptive biodiversity enterprise into a powerfully predictive one [35–37]. A major challenge is, therefore, catalyzing the digital mobilization and sharing of the massive but dormant biocollections data in institutions across Europe, North America, Russia, Brazil, India, and China.

### Challenge #2: Share expertise globally

A corollary to ‘flattening’ [sensu 38] the biodiversity science world is the desperate thirst for more information, tools, knowledge, and conceptual frameworks. That is, as science communities develop and begin to thrive in the developing world, increasing numbers of students and researchers are eager to learn the newest techniques and frameworks. Despite these advances and growing opportunities, most expertise currently still resides in Europe and North America.

Therefore, global sharing of skills in systematics and biodiversity informatics is a requisite step for true globalization of the community and the science. Without such training and expansion of the user community, the science and policy potential of increasingly available data will go unexplored, particularly in the developing world—the geographic areas of greatest biodiversity and environmental concern.

Capacity-building and training opportunities, in the narrow sense, are only important in the shortest term. Rather, we contend that this new, ‘flat’ world of biodiversity science demands full educational opportunities for students from developing countries, equivalent to those in the developed world, i.e., the opportunity to complete a doctoral program in research and education. Where these opportunities have opened, developing countries have become leaders in biodiversity information management: South Africa with SANBI [39], Mexico with CONABIO [40], Colombia with Instituto von Humboldt [41], Costa Rica with INBio [42], Brazil with CRIA [43], and India with several initiatives (e.g., India Biodiversity Portal; http://indiabiodiversity.org/). Scientists at these institutions have tackled and solved complex problems of assembling, maintaining, and sharing large biodiversity databases, and routinely perform sophisticated analyses that provide the science underpinnings of policy. In turn, these institutions now have the capacity and capability to develop high-level training programs that formerly depended on North American or European leadership. This model is the good virus of biodiversity science: at the moment, programs that spread it are a cottage industry, when what is needed are industrial strength solutions.

### Challenge #3: Avoid silos and reinvented wheels

A major challenge in biodiversity science is the degree to which information ‘silos’ are constraining integrative networks and deep insights. Quite simply, diverse data realms do not talk to one another very easily, as we pointed out in a recent review of the big questions in biodiversity informatics [1]. An excellent example is integrating the data that document and connect genome composition with the data that document species’ geographic occurrences, which is critical to elucidating insights into drivers of speciation and diversification [44]. Formats and protocols for persistent individual record identifiers have been developed that would greatly facilitate such crosstalk and integration, but they are not broadly available in either the geographic-occurrence data world (e.g., GBIF) or the genomic-data world (e.g., GenBank). As a consequence, the two data realms remain as distinct islands of data. To be linked and related, data about individual organisms represented in both realms must often be analyzed by hand. Initiatives to connect the real biotic data realms of genomes and geographic occurrence [45, 46] require a massive boost.

More broadly, new initiatives in biodiversity science frequently wave the flag of innovation and synthesis, but in the competitive game of identity politics, turf, and science funding, each initiative is effectively siloed from other such projects, sometimes on purpose, and sometimes for lack of broader vision of the importance of cross-linkage. As a result, the wheels of biodiversity science—standards, tools, data schemas, and structures, etc.—are re-invented, without benefit to the advancement of the field (see, e.g., the discussion of PIDs by Hardisty and Roberts, when a major evaluation has been developed recently [47]). Indeed, in some instances, such re-invention has not resulted in competitive vigor but a growth in biodiversity’s Babel—the non-interoperability of a plethora of data and analytical systems.

Most importantly, perhaps, biology lacks an underlying ‘unified theory of biodiversity’, and must rely on more local component frameworks, such as theories of natural selection and molecular evolution, ideas from island biogeography, etc. A broad, overarching theory would provide both the coherence and scaffolding on which to assemble and link the many entities of biodiversity information—molecular, physiological, morphological, systematic, ecological, phylogenetic, and spatial. Achieving this grand synthesis, however, is severely hampered by disciplinary and data silos; indeed, even exploration of component frameworks is hindered by lack of linkages among silos.

### Challenge #4: Deal with biodiversity science development challenges at the appropriate scale

The challenge of understanding biodiversity is neither regional nor global, but highly multiscalar—a network of local challenges that sums to a global-scale enterprise that must be engaged on multiple levels [48]. As a corollary, all aspects of this enterprise—building data resources, protocols, and human resources in biodiversity science—should also be multiscalar.

This principle is precisely why building local capacity and institutions are indispensable components of the biodiversity enterprise. In biodiversity science, local questions, perspectives, values, and approaches are as critical to success at that scale as are regional or national issues at those scales. Indeed, the work of biodiversity scientists and the education and training of students occurs at everything from local to global scales. This multiscalar property of biodiversity science belies the more geographically narrow view of Hardisty and Roberts [2].

We contend that solutions to the challenges described above and in Hardisty and Roberts [2] can be found in multiscalar approaches. For instance, digital capture and mobilization of the world’s biocollections data should be designed and implemented around resource partnerships between developing-world scientists, students, and institutions, whose biodiversity mandates often depend on acquisition of such data, and developed-world institutions equally eager to bring these data to the forefront of biodiversity research and synthesis. Such collaboration maximizes purpose, personnel, protocols, and institutional resources in meeting a daunting challenge.

Similarly, whereas GBIF has just passed the monumental mark of half of a billion records served via its data portal, too many of them are not adequately fit for use, particularly in lacking georeferencing. This challenge aches for a distributed global consortium of partners with expertise, tools, and experience in georeferencing data associated with biodiversity records, each partner being most knowledgeable about and committed to improving the data from its respective region. Global entities, perhaps even GBIF, could integrate and coordinate the effort, knowing that the geographic knowledge needed for broad and effective execution of this initiative is inherently regional or national.

In sum, although a truism, it bears repeating that solutions to challenges in biodiversity science require efforts at the appropriate scale, whether global, national or local, and often collaboration among entities at different levels. For example, a local issue will require coordination and implementation at that level, with funding and political will at national and regional levels [49]. This point is precisely the reason why the training of national and regional scientists, as well as local cadres (park managers, guides, rangers, etc.) is a *sine qua non* of biodiversity management.

### Challenge #5: Find strategic solutions

Goals cast so generally as to be unachievable are not particularly useful. In this sense, broad, overarching recommendations and targets that largely repeat initiatives already underway [2] are puzzling. Instead, setting limited, achievable goals with built-in rewards of accomplishment, significance, and impact will be much more strategic. As such, goals in biodiversity informatics, once reached, should bear near-term, exciting, and novel fruit.

**Figure 2:**
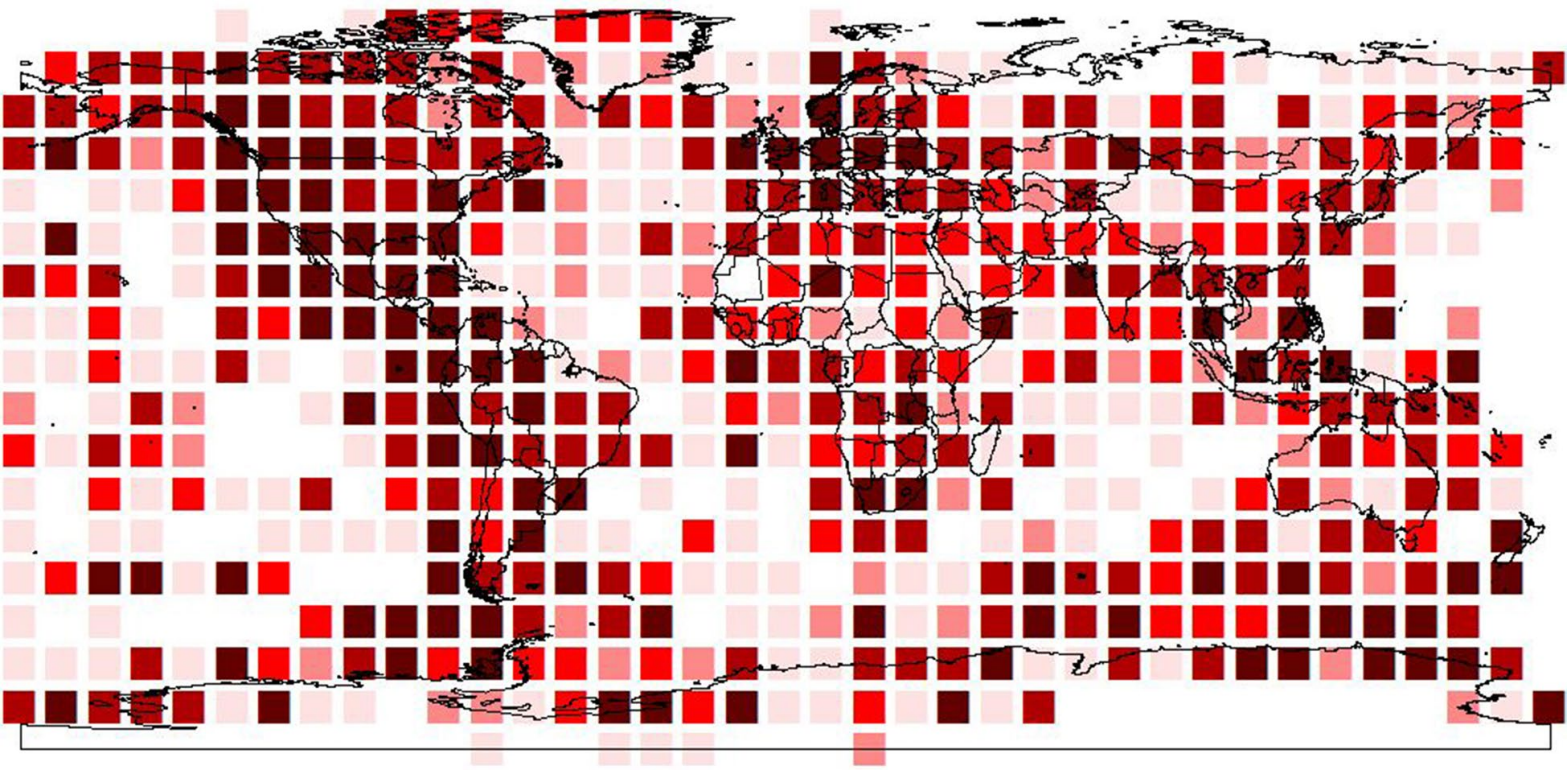
Global summary of completeness of knowledge of birds of the world at 10° spatial resolution. *White* none of avifauna documented, *darkest red* avifauna completely documented. From Peterson et al. (in prep.).

For example, accumulating biodiversity information by convenience rather than explicit strategy will build the absolute number of records served, but at the severe expense of mere quantity over quality, i.e., fitness for use for biodiversity science [27, 28, 50]. An example was the goal set by GBIF some years ago of serving one billion biodiversity records by 2010. Rather, a different, multipronged strategy would begin with a comprehensive gap analysis of existing biodiversity data. One prong might be to complete the coverage of groups that are already well-represented and near-comprehensive (e.g., birds; Figure 2), which would provide a complete view of known diversity in a single group. In parallel, other prongs would address remaining taxa according to explicit criteria, protocols, and lessons learned.

## Conclusions

Our decadal view of biodiversity informatics stands in sharp contrast to that of Hardisty and Roberts [2]. To be fair, we acknowledge the political and funding realities of European science, and Hardisty and Roberts [2] are at least explicit in their exclusive focus on Europe’s next decade. Nevertheless, the Hardisty and Roberts [2] paper is a useful cipher for the thinking and ills that pervade the field more broadly, which manifest in regional (not global) thinking and activity.

Unlike Hardisty and Roberts [2], our decadal view deliberately leapfrogs the well-worn points and priorities of the past decade or two, all of which were repeated as almost mantric recitations at innumerable meetings the three of us have attended. Instead, we focus our view on the fast-evolving global scientific and social landscape, which will govern the next generation of advances in biodiversity informatics. This landscape is increasingly being flattened and more evenly populated with scientists, students, institutions, initiatives, and data resources in countries that previously were considered scientifically underdeveloped. This flatter world is a powerful selective agent armed with big challenges and opportunities. Biodiversity science must adapt and adjust. Those sectors that won’t, will see its world sweep on by.

### **Authors’ contributions**

All three authors contributed equally to the development of this paper. All authors read and approved the final manuscript.

### **Acknowledgments**

We thank our many colleagues in biodiversity informatics for rich discussion and debate over many years, although they may not agree with much of what is said in this manuscript.

### **Compliance with ethical guidelines**

**Competing interests** The authors declare that they have no competing interests.

## Response

By Alex Hardisty

E-Mail: hardistyar@cardiff.ac.uk

Address: Cardiff University, School of Computer Science and Informatics, Queens Buildings, 5 The Parade, Cardiff CF24 3AA, UK

Our article [2] set out (for the first time, to our knowledge) a decadal view of challenges and priorities presently facing practitioners in biodiversity informatics. We presented a range of actions necessary to link the extensive array of available computerised resources and tools into a commonly-shared sustainable e-Infrastructure supporting all aspects of biodiversity and ecosystems science. We were explicit in saying we had considered the topic mainly from the European perspective. We provided a rallying point for community efforts, mainly in Europe it has to be said. We offered a baseline against which funding agencies could, if they choose assess new informatics proposals. However, we also said the vision is of global interest and relevance. The views were the result of a public consultation involving some 75+ contributing respondents, not all from Europe. On behalf of those contributors I’m grateful for the further correspondence by Peterson et al., which provides welcome additions to an important debate.

Biologists, ecologists, taxonomists, technologists and informaticians have to communicate and interact together. Only together as a global community can we achieve the right, interoperable, common informatics solutions to assist the science to generate the knowledge of how the biosphere works. Predicting the biosphere and providing sufficient evidence to manage it robustly is a greater challenge still. But, if we want to be able to do this in a scalable way, there are as Purves et al. [51] point out huge challenges to building useful models; not least in obtaining the appropriate types of data to validate the model predictions.

Data mobilisation, built on foundations of acquisition, whether by digitisation or other means; curation and preservation; discovery and open access; and ability to process; with inter-linkages and names playing their central roles is thus an essential strategic goal but one that has to be expressed as being for the explicit purpose. In this we can draw lessons from meteorology in the 1950s, 1960s and 1970s [52] where the purposes of geopolitics (nuclear arms race, and being first to put a man on the moon) were served with prioritised funding for meteorological models and supercomputing, and scientists collaborating together. This was not only to develop the models but also to identify and close data gaps and to re-work/invert the existing data. They “made global data and they made their data global”. Today that modern data, collected almost continuously around the world and the models that rely on it have significant commercial as well as scientific value for all kinds of stakeholders.

Essential biodiversity variables (EBV) [53] or similar indicators are a parallel case and a core future business; potentially with high scientific and commercial value that demands removal of barriers to global interoperability [54]. Just like weather variables, EBVs imply the ability to measure and calculate for any geographic area, small or large, fine-grained or coarse; at a temporal scale determined by need and/or the frequency of available observations; at a point in time in the past, present day or in the future; at appropriate scale, for any species, assemblage, ecosystem, biome, etc.; using data for that area/topic that may be held by any and across multiple data resources; using a standardised and widely accepted workflow capable of executing in any research infrastructure; and by any person anywhere.

What we see today in biodiversity informatics is, to use terminology from the article, mainly a “cottage industry”; or worse a subsistence economy with pockets of cottage industry. In the era of global societal challenges, global cooperation and a flatter world we need to make the transition to industrial-grade solutions. We need to work collectively, engaging with industry such that biodiversity/ecology professionals and industry together improve the way computer systems share, utilise and process information for biodiversity science. We must promote the coordinated use of standards we already have, and identify and adopt or develop those new ones still needed. We must mobilise the data to serve the purpose, rather than mobilising for mobilising sake. This creates interoperability benefits for the sector overall and profit opportunities to stimulate industry interest. Lessons from other sectors (healthcare for example [55, 56]) can show us how to tackle the issue.

Responding to some of the specific points in the article:

1. Peterson et al. are concerned that we are northward looking and almost exclusively European. As noted, we were explicit about the European perspective but the main themes of the vision [integration of available resources; support for scientific synthesis; a shared maintained multi-purpose network of computer-based data and processing services using a small set of (global) interchange standards] and the details needed to realise these themes are relevant in all corners of the world. This view is borne out by results from the international coordination project, CReATIVE-B working towards a global virtual environment for biodiversity research in its roadmap [54]. An international High Level Stakeholders Group comprising representatives of biodiversity and ecosystems research infrastructures from around the world serves to promote policy liaison and recommendations and coordinate towards that aim. The recently funded GLOBIS-B project to further coordinate informatics work to support EBVs, has support also from Australia, Brazil, China, South Africa, USA as well as Elixir, GBIF and GEO BON.

2. I see the alternative view and the five challenges offered by Peterson et al. not as a competing vision that “stands in sharp contrast” to our own but as a re-stating of or complement to what we propose. The issue is not that work remains to be started in all the areas we suggest nor that technical solutions still need to be found. Instead, it is that the works in progress need to become more widely known, to consolidate, to converge, and to embed in everyday practice right across the community. In this sense our vision is concerned much more with promoting infrastructure emergence and community consensus to achieve widespread buy-in, adoption and usage, than it is about solving any particular technical problem. We need to move more towards sustained funding anchored in pay-per-use or institutional commitments than to continue current hand-to-mouth dependencies on externally funded short-term projects.

3. Peterson et al. conclude with talk of leap-frogging, and I have some sympathy with that view. They evoke the fast-evolving, flatter more populous world of multiple stakeholders and encourage us to adapt to it or die. They ask for strategic solutions situated in this new world order and are right to do so but they do not offer the alternative scenarios that could play out in it. Without these we cannot yet find the best path to pursue for the most likely circumstances or more likely, for several different circumstances. We need to increase our depth of understanding by application of horizon scanning, scenario building and multi-path mapping techniques [57]. As should be clear by now, it is not the biodiversity informatics research that is the concern but the matter of how to translate results from that into everyday industrial-scale practice. Education and training curricula have an important role to play there as the authors have suggested but so does involvement of commerce/industry. I see with the hindsight of 2 years and from this perspective that our vision has not sufficiently addressed these and other sociological issues. Indeed in my own work establishing the Biodiversity Virtual e-Laboratory (BioVeL) infrastructure [58, 59] I see the new interest coming from eager young researchers outside of the established G8 and other western countries. However, I often ask myself whether we really sufficiently understand from the sociological and psychological perspectives how the complex technologies and methods we invent become effectively translated into practice. More work is needed.

In conclusion, I am happy that Peterson et al. have taken the time not only to read the original article but also to think about the issues and to write a response. I thank them for that and hope that such correspondence serves to further stimulate the debate and the consensus global action that has to follow. This is essential if modern biodiversity science, ecology and Earth stewardship are to fully benefit from the capabilities that informatics solutions offer.
